# Leukopenia, weight loss and oral mucositis induced by 5-Fluorouracil in hamsters’ model: A regenerative approach using electrospun poly(Lactic-co-Glycolic Acid) membrane

**DOI:** 10.18632/oncotarget.28685

**Published:** 2025-02-18

**Authors:** Ana Chor, Hélio dos Santos Dutra, Marcos Lopes Dias, Raquel Pires Gonçalves, Alexandre Malta Rossi, Christina Maeda Takiya, Marcos Farina

**Affiliations:** ^1^Biomineralization Laboratory, Institute of Biomedical Sciences, Federal University of Rio de Janeiro, Rio de Janeiro 21941-902, Brazil; ^2^University Hospital, Bone Marrow Transplantation Unit, Federal University of Rio de Janeiro, Rio de Janeiro, 21941-902, Brazil; ^3^Catalysis Laboratory for Polymerization, Recycling and Biodegradable Polymers, Institute of Macromolecules Professor Eloisa Mano, Federal University of Rio de Janeiro, Rio de Janeiro 21941-598, Brazil; ^4^Department of Condensed Matter, Applied Physics and Nanoscience, Brazilian Center for Research in Physics, Rio de Janeiro 220-180, Brazil; ^5^Immunopathology Laboratory, Institute of Biophysics Carlos Chagas Filho, Federal University of Rio de Janeiro, Rio de Janeiro 20941-902, Brazil; ^*^These authors contributed equally to this work

**Keywords:** leukopenia, weight loss, oral mucositis, PLGA dressing, regeneration

## Abstract

Clinical parameters of leukogram and weight were analyzed in animal models before and after seven days of 5-FU infusions. A comparison of leukograms before and after 5-FU administrations was analyzed. The results showed a significative difference (*p* = 0,004), confirming the immunosuppression. There was a decrease in the weight of the animals after 7 days of 5-FU infusions (*p* = 0.02). After immunosuppression, oral mucositis (OM) ulcerative lesions were established. Two of ten animals were aleatory selected to receive PLGA dressings. The electrospun PLGA membranes with or without autologous cells were applied to the ulcerative lesions, aiming to accelerate the regeneration process. Although this therapeutic innovation for OM lesions was still not tested in the bioengineering area, morphological analysis presented promising results. Lesions covered by cell-free PLGA exhibited areas of inflammation persistence and angiogenesis, and cell-seeded PLGA membrane exhibited complete reepithelialization after 6 days, with minor inflammatory infiltrate. Interestingly, the present work showed preclinical parameters of cachexia induced by chemotherapy for cancer treatment. Moreover, it showed an innovative approach by applying dressings constituted of electrospun PLGA with the addition of autologous mesenchymal cells for OM ulcerative lesions. This promising innovation will pave the way for future applications in oral mucosa lesions.

## INTRODUCTION

The 5-Fluorouracil (5-FU) is an antimetabolite that promotes DNA damage by inhibiting thymidylate synthetase (TS), and depleting thymidine triphosphates (TTPs) available for DNA synthesis [1]. 5-FU can be incorporated into DNA and RNA in the region of thymine or uracil, respectively [2]. The incorporation of analogs of purine and pyrimidine bases into DNA during the S phase of the cell cycle prevents the addition of nucleotide bases and causes DNA replication failure. [2, 3]. Leukopenia is a hematological toxicity induced by 5-FU, a first-choice chemotherapy agent for several cancers. Furthermore, weight loss, oral mucositis, and diarrhea associated with the same agent, are parameters that influence quality of life and treatment outcomes during cancer treatment [4]. However, it is important to highlight that weight loss has been analyzed as a parameter for cachexia along cancer treatment, which increases muscle and adipose tissue loss leading to weakness and fatigue [4].

Serious complications can arise from oral and gastrointestinal mucositis caused by the antimetabolite 5-FU during head and neck cancer treatment, due to disruptions in soft tissues. [5]. On the other hand, intensive association of other chemotherapy agents can culminate in deleterious effects, wich can result in serious OM lesions. At this moment, the deleterious side effects interfere with the patient’s compliance with treatment and the outcomes. [6]. Despite better survival rates after cancer treatments, several therapies may increase the percentage of patients with OM to approximately 90%–100%. [5, 7, 8]. However, due to a lack of effective treatments for OM lesions, there is an increase in investigations using animal models. These studies aim to explore new therapeutic approaches that either disrupt the pathways leading to OM lesions, or accelerate their healing process. [9–11]. Chor et al., [12] discussed the molecular pathways of OM in detail, and a range of emerging approaches in preclinical models, with the potential for future clinical applications. Interestingly, natural products like honey or olive oil have been explored to treat chemotherapy-induced OM in children with leukemia [13]. Metformin, an antidiabetic drug, was explored to treat chemotherapy-induced effects in non-diabetic breast cancer patients [14]. Zinc has recently been used to prevent OM in children with cancer receiving intensified chemotherapy [15]. Topical Omega-3 nanoemulgel was recently explored for radiotherapy-induced OM in patients with Head and Neck cancer [16]. And, Collela presented a range of therapies that significantly reduced the severity and incidence of oral mucositis. [5].

Biomaterials used in tissue engineering and regenerative medicine have been a promising therapy for OM lesions in preclinical and clinical trials. [11, 12, 16, 17]. One of the trends in the bioengineering area is the use of natural or synthetic three-dimensional (3D) biodegradable scaffolds as a biomimetic strategy. This strategy aims to reproduce the natural extracellular matrix, and to promote an ideal microenvironment for cell adhesion and proliferation [18]. Notably, the electrospinning is a fiber-forming process to produce scaffolds that mimic the human organism. This technology is booming in the research and industrialization of advanced fiber-based materials [19]. The electrospinning machine can be set up to produce fibers in various forms and with distinct morphologies. A diversity of fiber-formed materials have been applied for tissue engineering [20–22], drug delivery [20], wound dressing [22], biotechnology [23], and others [19]. An example is the electrospun polylactic-glycolic acid (PLGA), whose starting materials are biocompatible monomers, such as lactic and glycolic acids [24]. The physicochemical analysis of PLGA was extensively explored by our group, owing to its biodegradability and good mechanical for tissue engineering. [24]. In addition, Chor et al., [25] previously explored Madin-Darby canine kidney cells (MDCK) lineage, and fibroblast-like cells from hamster cheek pouch onto electrospun PLGA (85:15) membranes [25]. Those cells expanded into the membrane fibers and formed a cluster of cells among the interconnected fibers and pores over time [25].

The advancements in tissue engineering and regenerative medicine opened ways for a variety of biomaterials’ blend taken by different processes [18]. Such materials have been successfully applied in the regenerative process of hard and soft tissues of various organs, and also in the oral cavity tissues [26, 27]. The present work explored the adverse effects of leukopenia and weight loss during 5-FU infusions in hamsters’ models, which resulted in OM lesions. Moreover, the present work delved into the foundations of tissue engineering and regenerative medicine area, to explore a pioneering therapy for OM ulcerative lesions, with the potential of treating other tissue injuries in future studies. [28].

## RESULTS

### Comparison of leukograms before and after 5-FU or saline solution administrations

**Figure 1:**
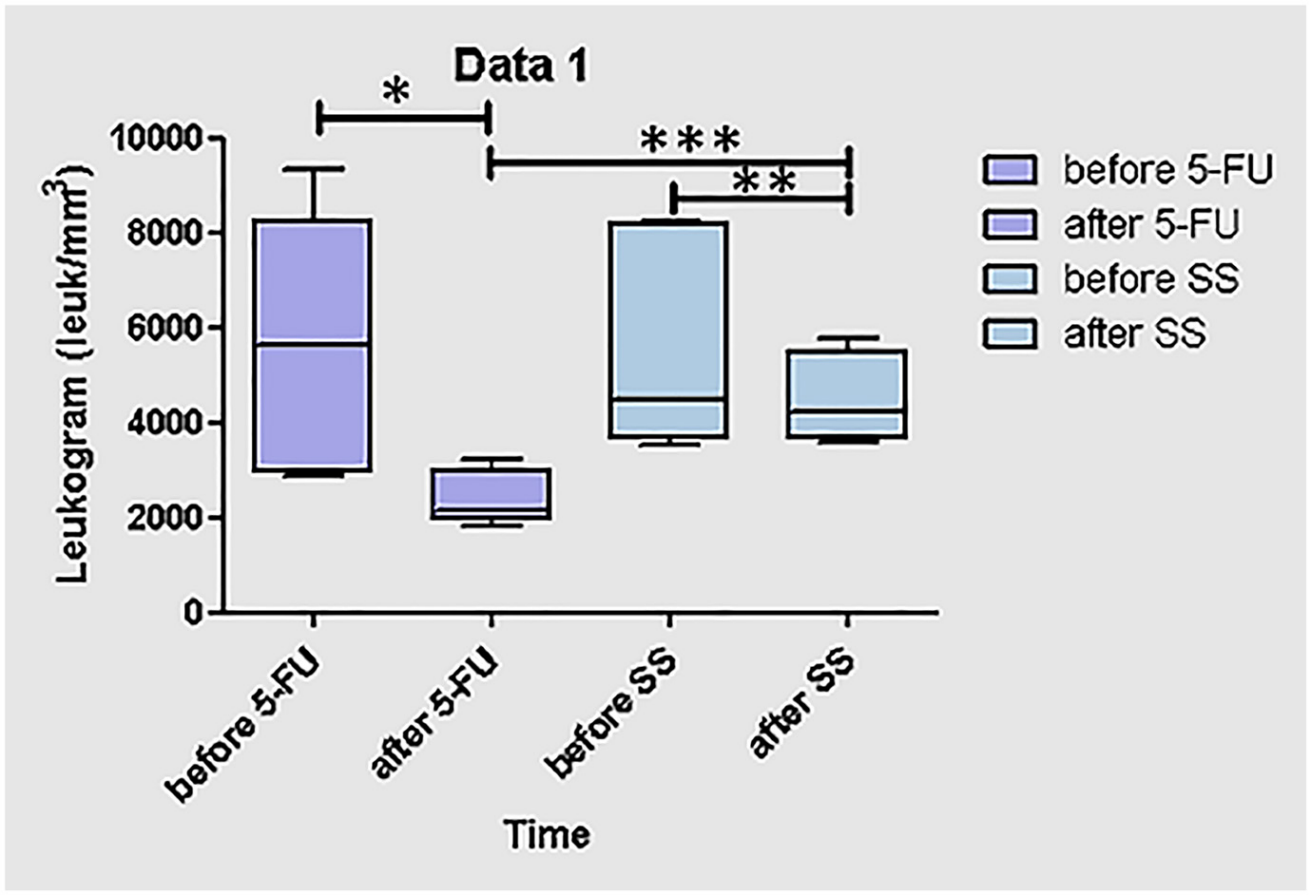
Representative graphic of leukograms of the population of animals treated with 5-FU or saline solution as control. (^*^) Leukogram before and after 4 days of 5-FU infusion, analyzed by Wilcoxon test, characterizing comparisons between independent leukograms of the same population, paired to be analyzed in different times (*p* = 0,03); (^**^) Leukogram before and after 4 days of saline solution infusion, analyzed by Wilcoxon test, characterized comparisons between independent leukograms of the same population, paired to be analyzed in different times, exhibit no significative results (*p* = 0,222); (^***^) Leukogram before and after of 5-FU or saline solution infusions, analyzed by Mann Whitney Test, characterized the comparisons between independent leukograms from independent treatments, in different populations of animals at the same time (*p* = 0,04). The *p* < 0,05 value was considered the reference of the statistical hypothesis.

The results of the comparison between the leukograms, before and after 5-FU administration, showed significant results related to immunossupression after the first 5-FU infusion (Figure 1, *p* = 0,03). Represented by the lower and higher values of the medians before 5-FU (287,5–935,0), and after 5-FU (185,0–322,5) infusions, Table 1. The results of the comparison between the leukograms before and after saline solution (SS) administration, showed no significant results related to immunossupression after the first SS infusion (Figure 1, *p* = 0,222). Represented by the lower and higher values of the medians before (355,0–825,0), and after SS (360,0–577,5) infusions, Table 1. Another analysis characterized the comparisons between independent leukograms from independent treatments, in different populations of animals at the same time (Figure 1, *p* = 0,04), 4 days after 5-FU or SS administrations. The *p* < 0,05 value was considered the reference of the statistical hypothesis.

**Table 1:** Medians corresponding to the lower and higher values of leukogram in each population of animals

Measures	Before - 5-FU/mm^3^	After - 5-FU/mm^3^	Before - SS/mm^3^	After - SS/mm^3^
Lower	2875,0	1850,0	3550,0	3600,0
Median	5650,0	2123,0	4500,0	4250,0
Higher	9350,0	3225,0	8250,0	5775,0

### Comparision of independent weight loss of 5-FU and saline solution (SS) groups of animals

**Figure 2:**
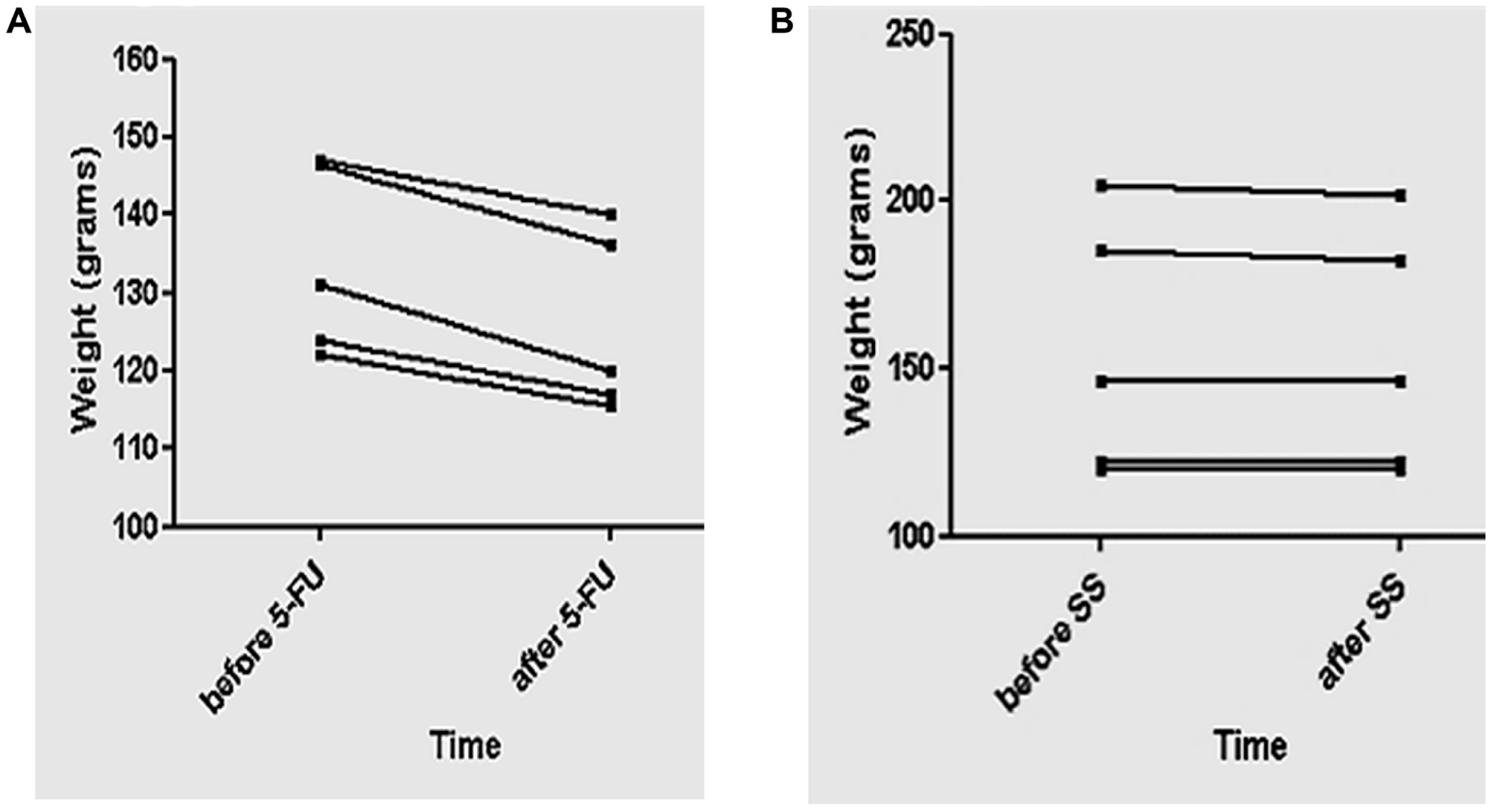
Representative graphics of the animals’ weight treated with 5-FU or SS as control. (**A**, **B**) The representative graphic exhibit the analysis related to the weight before and after 4 days of 5-FU or SS administration, using the paired *t*-test. The analysis is showing comparisons between independent weights at the same population of animals, pared to be analyzed in different times. Graphic A is showing that 5-FU induced weight loss, revealing significative difference (*p* = 0,02). Graphic B is showing comparisons of independent weights in the same population of animals treated with SS, exhibiting no significative statistical difference (*p* = 0,19). The value of *p* < 0.05 was the reference for the hypothesis of statistical difference.

The weight of the population of animals with distinctive modalities of treatments were analysed (Figure 2A, 2B).

The comparison of the weights of the animals before and after 5-FU infusion revealed significant results (Figure 2A) (*p* = 0,02) related to weight decrease, which was represented by the minimum and maximum values of the medians before (122,0–147,0), and after 5-FU (115,5–140,0) infusion, Table 2. On the other hand, graphic B (Figure 2B) exhibits comparisons of independent weights in the same population of animals, before and after saline solution (SS) infusion. The results showed no significant statistical difference related to weight decrease (*p* = 0,19), represented by the minimum and maximum values of the medians before (120,0–205,0), and after SS infusion (120,0– 202,0) Table 2. However, decreases were observed in two animals. The *p* < 0,05 value was considered the reference of the statistical hypothesis.

**Table 2:** Medians corresponding to the lower and higher values of weight in each population of animals

Measures	Before - 5-FU/mm^3^	After - 5-FU/mm^3^	Before - SS/mm^3^	After - SS/mm^3^
Lower	122,0	115,5	120,0	120,0
Median	131,0	120,0	146,0	146,0
Higher	147,0	140,0	205,0	202,0

### Macroscopic image and histomorphological analysis of OM ulceration

**Figure 3:**
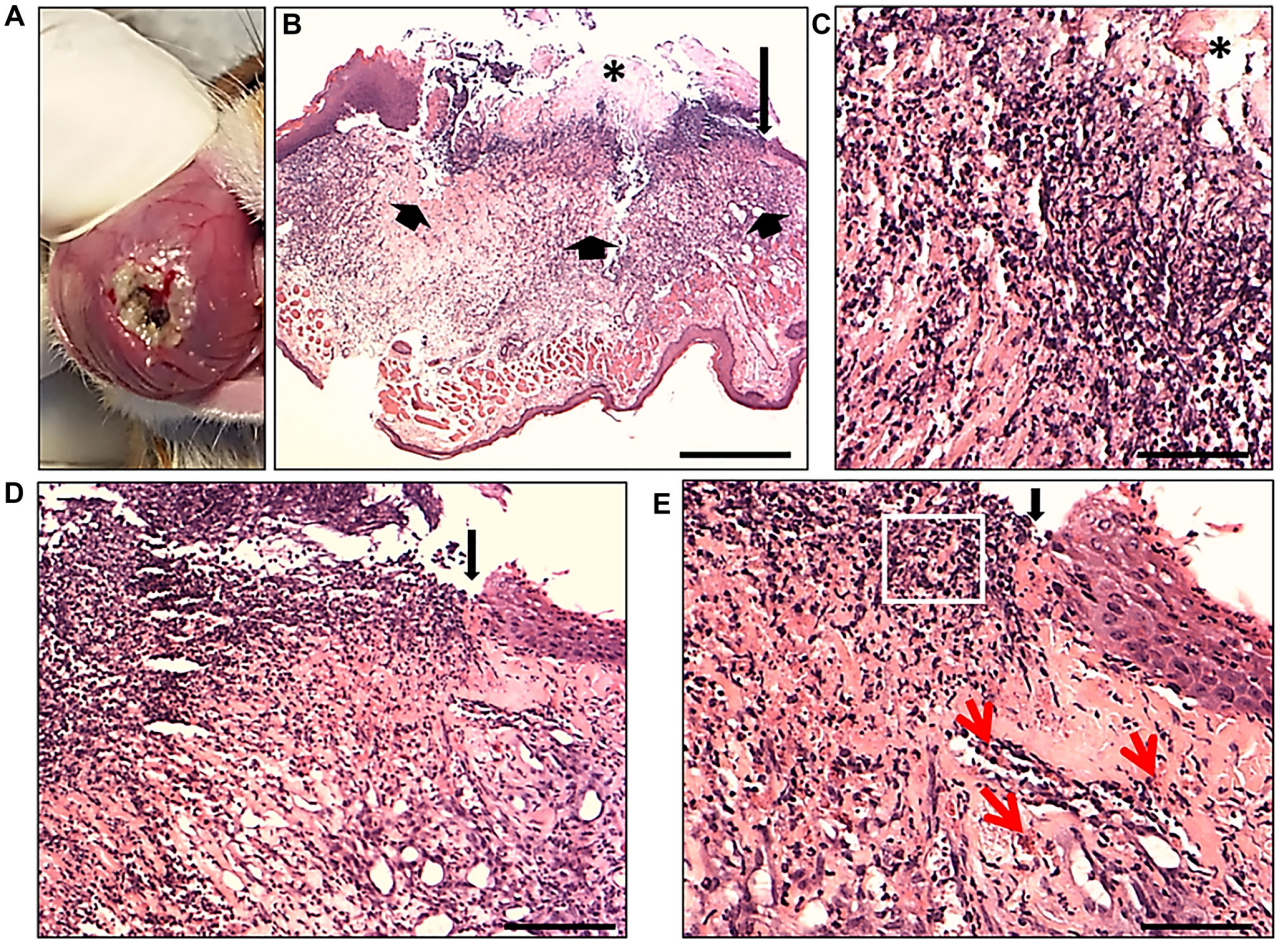
Representative micrographs of the macroscopic lesion of oral mucositis and histology of the OM ulceration produced in an experimental hamster model 7 days after 5-FU administration and scratch. Macroscopic view of OM ulceration establishment, 7 days after administration of 5-FU and scratch procedure (**A**). Note the ulcer with a crateriform aspect, everted edges and apparent necrotic tissue, characterizing a pseudomembrane, surrounded by hyperemic area of mucous tissue with vessels and hemorrhagic areas. (**B**) Full histological view of ulcer formation (short arrows) induced by 5-FU and scratch, with intense mononuclear inflammatory infiltrate (inflammatory phase). The presence of cellular debris and a zone of necrosis (^*^) on the ulcer was exhibited, characterizing the pseudomembrane; above, the long arrow shows a region of epithelial discontinuity; scale bar: 500 μm. (**C**) A greater magnification of the same region observed in (B), shows a detail of the necrosis zone (^*^) (pseudomembrane) with predominant inflammatory infiltrate; scale bar: 100 μm. (**D**) Higher magnification of the same region in (B) (long arrow) showing an ulcerated area with cellular debris and the presence of an epithelial tongue at the end (regenerative epithelium) can be seen (after the arrow); scale bar: 200 um. (**E**) At a higher magnification of the same region in (D), there is evidence of inflammatory cells in the ulcer region (square) and vessels in the stroma (red arrows), as well as the presence of an epithelial tongue at the extremity (regenerative epithelium after the black arrow); scale bar: 100 μm. HE stains. Eclipse E800 light microscope (NIKON, Japan).

Figure 3A depicts the macroscopic view of 5-FU-induced OM ulceration in hamster model, exhibiting the ulcer formation with a crateriform aspect, everted edges and apparent necrotic tissue, characterizing a pseudomembrane surrounded by hyperemic areas of mucous tissue with vessels and hemorrhagic areas. In the histomorphological analysis (Figure 3B–3E); Figure 3B represents the histomorphological analysis of the cheek pouch of the animal with 5-FU-induced OM ulceration (narrow arrows). The image depicts the ulcerative region with the disrupted epithelial layer (long arrow) showing the pseudomembrane formation represented by the necrotic area (asterisk). Figure 3C exhibits greater magnification of the same region observed in Figure 3B, showing a detail of the necrosis zone (^*^) (pseudomembrane), with predominant inflammatory infiltrate; Figure 3D depicts a higher magnification of the same region in (B) (long arrow) showing an ulcerated area with cellular debris and the presence of an epithelial tongue at the end (regenerative epithelium after the arrow); Figure 3E depicts a higher magnification of the same region in (D) showing the evidence of inflammatory cells in the ulcer region (Figure 3E, square) and vessels in the stroma (Figure 3E, red arrows), as well as the presence of an epithelial tongue at the extremity (regenerative epithelium after the black arrow).

### Macroscopic and histomorphological images of OM and regeneration after PLGA dressing with or withought the animal’s cells

**Figure 4:**
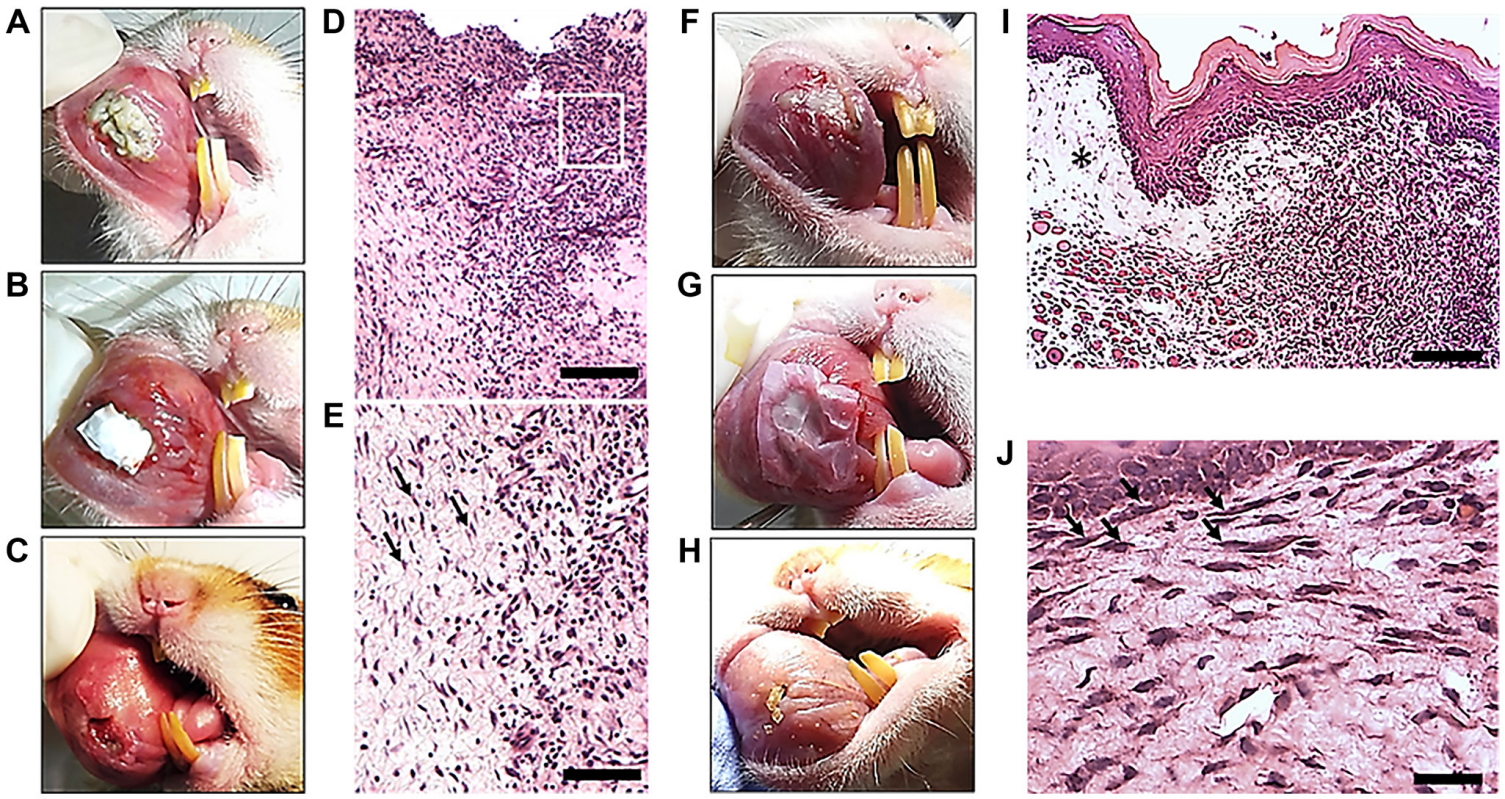
Macroscopic images of OM ulcerations after 7 days of 5-FU administration, and histomorphological analysis after PLGA dressings with or without the animal’s cells. Macroscopic view of OM ulceration induced by 5-FU and scratch (**A**). Note the necrotic area covering the ulcerated region in the jugal mucosa of the animal; (**B**) macroscopic view of PLGA dressing without cells covering the ulcer; (**C**) macroscopic view of OM ulceration 6 days after PLGA dressing without cells. Note the amelioration of the ulcer, still with tissue hyperemia and visualization of the borders of the ulcer; (**D**) histomorphological image of OM ulceration 6 days after 5-FU administration (bar: 200 µm). Note that there is no epithelial layer and the inflammatory infiltrate persists in the subepithelial region (square); (**E**) the same image of A showing the inflammatory infiltrate adjacent to the regenerative region exhibiting fibroblasts (arrows) (bar: 200 µm); (**F**) Macroscopic view of OM ulceration in another animal 7 days after 5-FU administration. (**G**) Macroscopic view of PLGA dressing with cells covering the ulceration; (**H**) macroscopic view of OM ulceration 6 days after PLGA dressing with cells. Note the ulcer regeneration in the jugal mucosa. (**I**) Histomorphological image of OM ulceration 6 days after PLGA dressing with the addition of cells (bar: 200 µm). Note the regenerated epithelial layer (white asterisks) and the newly formed tissue in the subepithelial layer (black asterisk). (**J**) The same image of (I) showing the proximity of fibroblasts to the basal layer of the epithelium (arrows) in the regenerated area (bar: 200 µm). HE stains. Eclipse E800 light microscope (NIKON, Japan).

The macroscopic view of OM ulceration iduced by 5-FU and scratch is represented in Figure 4A, showing the necrotic area covering the ulcerated region in the jugal mucosa of the animal. Next, Figure 4B shows the macroscopic view of PLGA dressing without cells covering the ulcerative area, and Figure 4C represents the macroscopic view of OM ulceration 6 days after PLGA dressing without cells. Note the amelioration of the ulcer, still with hyperemia area surrounding the remaining ulcerative region, exhibiting the borders of the ulcer. Figure 4D is showing the histomorphological image of OM ulceration 6 days after 5-FU administration. Note that there is no epitelial layer and the inflamatory infiltrate persistis in the subepithelial region (Figure 4D, square). Figure 4E represents the same image of Figure 4D showing the inflammatory infiltrate adjacent to the regenerative region, and fibroblasts (arrows). Figure 4F represents another animal with 5-FU-induced OM lesion, which was covered with PLGA membrane with the addition of the animal’s cells (Figure 4G). Next, Figure 4H is showing the macroscopic view of OM regeneration 6 days after PLGA dressing with cells. The regenerative area exhibits the jugal mucosa without ulceration. Figure 4I exhibits the histomorphological image of OM ulceration 6 days after PLGA dressing with the addition of cells, showing the regenerated epitelial layer (white asterisks) and the newly formed tissue in the subepithelial layer (black asterisk). Figure 4J exhibits the same image of (I) showing the proximity of fibroblasts to the basal layer of the epithelium in the regenerated area (arrows).

### Blood vassels and macrophages were observed in the interface of the regenerative area

**Figure 5:**
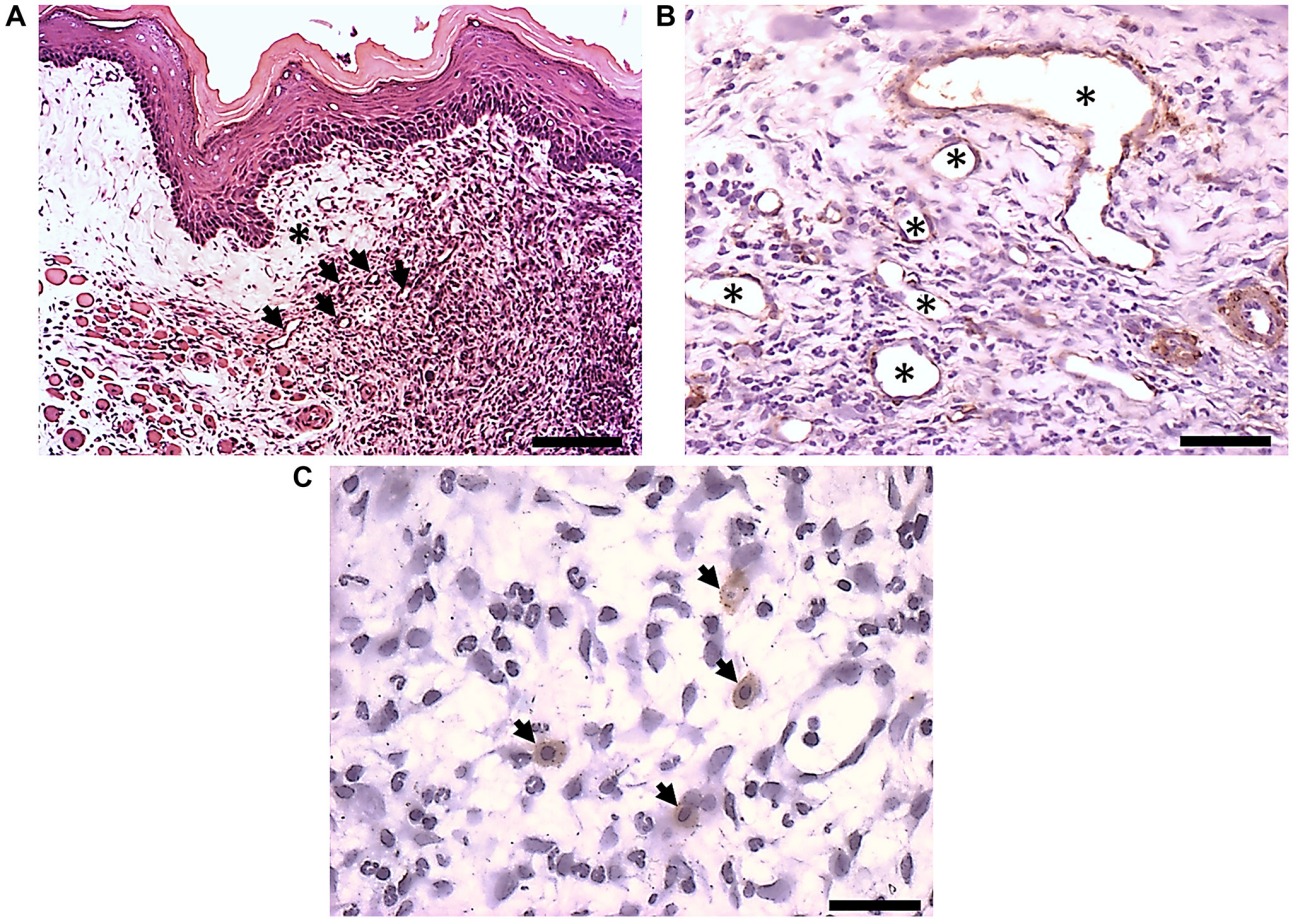
Immunohistochemistry of α-SMA and F4/80 positive cells. Detail of histological sections after PLGA dressing with the animals’ cells. (**A**) Histomorphological image of OM ulceration 6 days after PLGA dressing with cells (bar: 200 µm). Note the interface of the ulceration site (white asterisks) with the newly formed tissue in the subepithelial layer (black asterisk), exhibiting new formed vessels (arrows). (**B**) Detail of histological sections in the interface of the regenerative area showing α-SMA positive cells in the vessels walls (asterisks) (bar: 100 μm). (**C**) Detail of histological sections in the interface of the regenerative area showing F4/80 positive cells, exhibiting macrophages surrounding a blood vessel in the stroma, at the interface of the regenerative area (bar: 100 μm).

The histomorphological image of OM ulceration 6 days after PLGA dressing with cells is represented in Figure 5A. In the histomorphological image, the regenerative area (Figure 5A, black asterisk) was observed adjacent to the inflammatation site (Figure 5A, white asterisk) after PLGA dressing with the animal’s cells. In the interface of the regenerative area, newly formed blood vessels were observed (Figure 5A, black arrows), confirmed by α-SMA (α-smooth muscle actin) positive cells in the vessels’ walls (Figure 5B, asterisks). Also, F4/80 positive cells, (macrophages), were observed sorrounding the stroma in the interface of the regenerative area (Figure 5C, arrows).

## DISCUSSION

Leukopenia and weight loss are significant clinical side effects associated with the 5-FU agent used in the treatment of various cancers [4]. Cancer patients usually undergo chemotherapy in cycles, with each cycle involving a five-day infusion of the drug, which accounts for one week of treatment.

**Figure 6:**
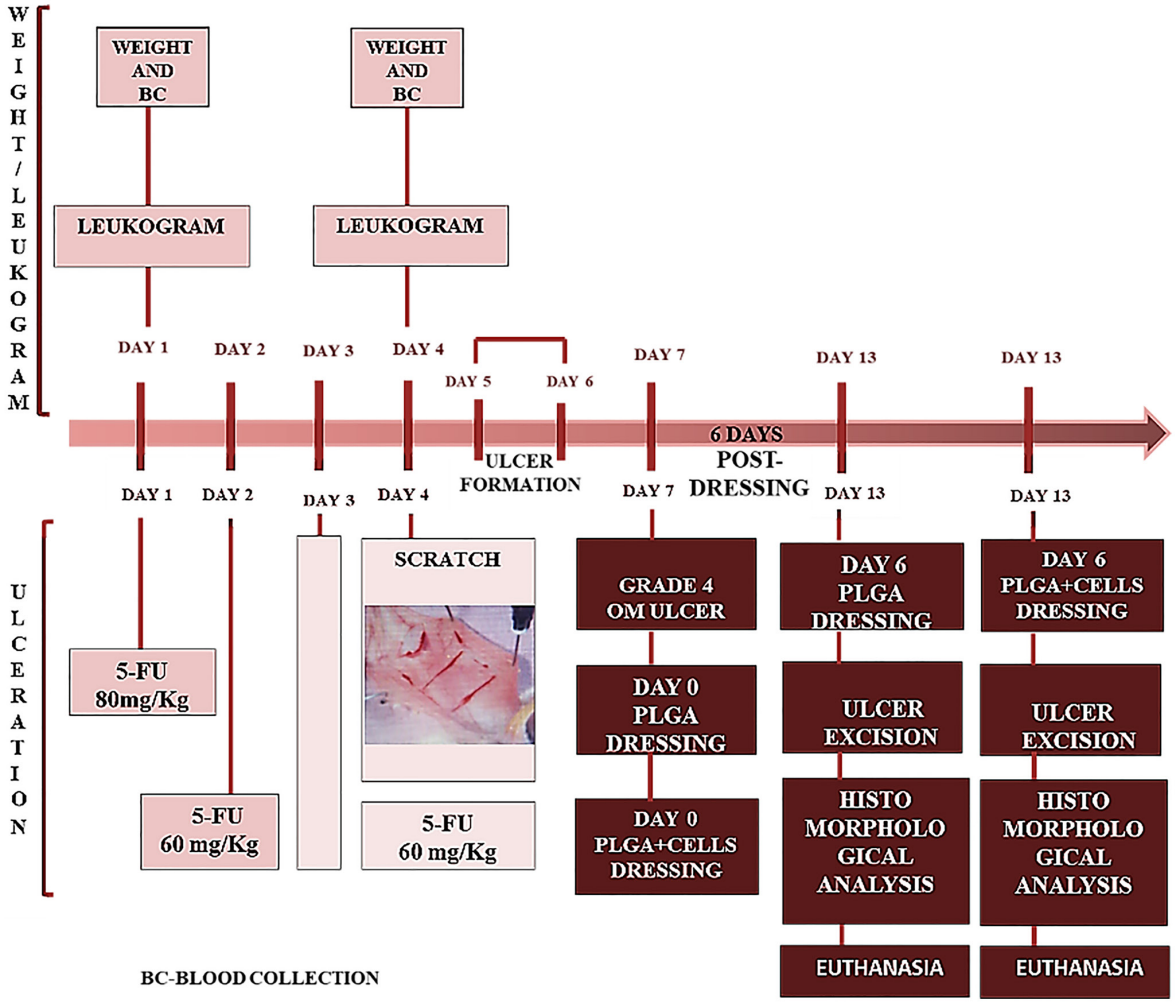
Timeline diagram presenting the *in vivo* experimental design. day 1: weight and blood collection for leukogram were performed before the first chemotherapy administration (5-FU/80 mg/Kg). day 2: second day of 5-FU administration (5-FU/60 mg/Kg); day 3: without treatment. day 4: weight and blood collection for leukogram was done before the third chemotherapy administration (5-FU/60 mg/Kg) and scratch on the hamsters’ cheek pouch. days 5 and 6: period of time for ulcer formation. day 7: establishment of grade 4 ulceration and treatment with PLGA dressing (day 0 for treatment initiation) with or without hamster’s autologous cells. day 13: represents 6 days post PLGA dressing with or without cells. Day of ulcer excision for histomorphological analysis (optical and electron microscopy) and euthanasia.

Recently, Vanderveen reported findings that align with initial subclinical signs of cachexia after just one cycle of 5-FU [4]. These results from mouse models demonstrated that a single cycle was sufficient to induce significant leukopenia and body weight loss. Additionally, there was a reduction in the total number of skeletal muscle immune cells and a decrease in certain inflammatory mediators [4]. In the present study, 5-FU was not administered over a cycle of five consecutive days (see Figure 6). However, the results demonstrated leukopenia and weight loss in hamster models after three days of 5-FU infusions. During antineoplastic treatment, cachexia is a common complication associated with chemotherapy, concomitant with the adverse effect of oral mucositis, due to metabolic homeostasis failures [29]. Those failures are associated with periods of treatment-dependent immune system suppression, resulting in decreased neutrophil counts and weight. In the present study, data on leukopenia and weight loss confirmed the establishment of immunosuppression, which resulted in 5-FU-induced oral mucositis. The leukogram of the animals treated with either 5-FU or saline solution was conducted prior to the initial chemotherapy or saline infusions, as well as before the procedure to induce oral mucositis lesions.

Immunosuppression was well observed in the overall reduction of the leukogram, but was not significant to demonstrate difference in weight between the populations of animals treated with 5-FU or saline solution. However, 5-FU promoted significant difference in weight loss. Our findings align with the study by Bertoloni and colleagues, who reported weight loss and neutropenia following intravenous 5-FU treatment for oral mucositis induction in experimental mouse models [30]. The authors reported significant findings regarding reductions in blood cell counts, confirming that immunosuppression was induced. Moreover, our findings are consistent with those of Lee and colleagues, who reported decreases in weight loss and neutrophil counts following intraperitoneal infusions of 5-FU for inducing oral mucositis in hamster models [31].

Due to the severity of oral mucositis induced by chemotherapy, our team has developed a groundbreaking preliminary approach using PLGA dressings. This method may or may not include the addition of autologous cells from animals. The primary goal of this innovative approach is to accelerate the healing process of ulcerative lesions associated with oral mucositis.

The implantation of a biomaterial in a host disrupts the homeostasis of the target tissue, potentially leading to molecular signals that trigger immune responses [32]. Although, the surface properties of a biomaterial play an important role in the modulation of immune responses, such as foreign body reactions [32], this reaction can be present in the tissue-material interface as long as the material’s fragments persists [32].

According to Siepmann [33], PLGA degrades when in contact with biological fluids, which results in shorter chain alcohols and acids. An accumulation of the latter can lead to significant drops in micro-pH and subsequent acceleration of polymer degradation. Our group previously explored the degradation of PLGA membranes in three important biological fluids for future oral mucosa regeneration [24]: immersions in simulated body fluid (SBF), culture media (Dulbecco’s Modified Eagle’s Medium – DMEM) and artificial saliva at 37ºC, for 7, 15 and 30 days were compared with the control membrane. In addition, Chor et al. [24], reported that electrospun PLGA 85:15 nonwovens were constituted by a semicrystalline material with moderate degradation properties after thirty days. The PLGA electrospun nonwovens presented a preferential degradation of the amorphous fraction of the material increasing the crystalline content along the time [24]. Regarding the kinetics of degradation after sterilization with gamma radiation, it was slower on non-irradiated samples than on irradiated ones, due to mass loss [24]. However, SBF showed a higher capacity of influencing crystallinity changes in both irradiated and non-irradiated PLGA nonwovens [24]. Fourier-transform infrared spectroscopy (FTIR) analyses revealed that the increased OH stretching modes and additional bands indicated that both SBF and saliva caused more significant hydrolytic degradation of irradiated PLGA nonwovens [24]. We monitored the fluid-induced degradation by *weight average molecular weight* (Mw) of PLGA nonwovens immersed over 30 days [24]. Effectively, our results indicated a trend of decreasing molecular weight in both non-irradiated and irradiated nonwovens, when immersed in various solutions, in comparison with the control samples [24]. This event is compatible with cleavage of the covalent bonds in the polymer backbone [24]. Although the recorded mass loss corresponds with the early stages of polymer degradation [24], its structure remained intact. This structure is essential for promoting optimal cell ingrowth and proliferation, as shown in our previous *in vitro* experiments. [25]. In this study, the promising results indicated that our manufactured membrane serves as an ideal scaffold for regenerative purposes [25], as demonstrated in the preliminary results reported here. Additionally, we previously investigated the implantation of electrospun PLGA membranes (1 cm² × 1 cm²) in the backs of hamsters over 7, 15, 30, and 90 days to observe tissue interactions with the manufactured biomaterial. [25]. The samples analyzed by transmission electron microscopy revealed a decrease in PLGA fragments 90 days after implantation [25]. The analysis processed seven days post-implantation revealed internalized fragments by epithelioid cells, without any signs of fibrosis or encapsulation of the biomaterial’s fragments [25]. Additionally, the chronic inflammatory response diminished by 90 days post-implantation, and also no fibrosis formation was observed. In addition, we observed improved vascularization [25]. Similarly, Klabucov [34] showed enhanced vascularization following biomaterial implantation. The researchers implanted electrospun polycaprolactone microfiber scaffolds with the addition of pCMV-VEGF165-plasmid in the subcutaneous interscapular area of rats over 7, 16, 33, 46, and 64 days. In this study, the observed vessel growth did not align with the rate of scaffold degradation [34]. Given polyesters’ autocatalytic degradation properties, we intentionally chose not to explore the linkage between cell physiology and the degradation process. This decision was influenced by our group’s previous findings, which showed remarkable cell proliferation and favorable cell morphology, showing a device with the potential for future tissue engineering applications. [25]. In this research work, we delved into the promising potential of cell-seeded membranes, with the addition of Mandy-Darby canine cell line, or primary fibroblasts from hamster cheek pouches onto PLGA membranes. Our findings demonstrated significant cell proliferation and enhanced cell migration through the membrane pores over time. Specifically, the primary fibroblasts showed changes in their morphology. They adopted a polygonal shape when attaching to the fibers that create the pores of the membrane, while they exhibited an elongated pattern when anchoring along a single fiber. Concerning, our earlier promising *in vitro* and *in vivo* results, we hypothesize that the autocatalytic degradation process of our semicrystalline scaffold did not influence cell proliferation rates. The membranes were manufactured using ratios of 85% PLA (polylactic acid) and 15% PGA (polyglycolic acid). This PLGA blend exhibited an initial degradation of the amorphous portion due to the higher content of the crystalline portion, that generally takes longer to degrade. In the present research work, the same electrospun PLGA membrane was used. Firstly, we planned to present our preliminary *in vivo* findings to our peers, as detailed here. As anticipated, our intriguing preliminary results motivated us to continue exploring future research works in the field of bioengineering.

Interestingly, in the present study, our group found that the PLGA dressing alone promoted repair and re-epithelialization in part of the lesion. However, it did not achieve strong adhesion between the epithelium and the connective tissue at the edges, and there was an area of inflammation. Conversely, when the PLGA dressing was combined with the animal’s cells, accelerated the re-epithelialization process, showing reduced inflammation and enhanced angiogenesis. The histopathological analysis revealed that macrophages were surrounding vessels in the stroma at the interface of the regenerative area, the PLGA dressing, and the surrounding cells, six days after cell-seeded PLGA membrane application. It is important to investigate the plasticity of macrophages, which can differentiate into two main types, characterized by distinct metabolic activities and phenotypic markers: M1 and M2 [35]. Given a classic division, activated macrophages (M1) are associated with acute inflammatory responses. However, alternative macrophages (M2) are associated with inflammatory lesions’ resolution [35]. The results of the morphological analysis reveled that macrophages are present at the interface of the regenerative area [35]. In this sense, we can hypothesize they are macrophages that have changed to an alternative phenotype M2 or antiinflammatory [36, 37], which promotes polymer phagocytosis and releases chemokines that induce angiogenesis and tissue repair [36, 38]. However, the M2 type is divided into four categories. The first one is related to foreign body reaction formation (M2-a type), and occurs when the immunologic system is exposed to a foreign material or organisms that cannot be engulfed or phagocytosed by a single macrophage [35]. Chor et al. [25] previously reported foreign body reactions after PLGA implantation in the back of Hamsters. Our results aligns with the first type of macrophage formation, since we did not observe fibrosis formation. In the present research work, we did not observe such reactions when using the same membrane as dressings for oral mucositis lesions. Due to the intriguing results, further research involving larger sample sizes is necessary to validate the preliminary findings. It is interesting to note that, the surgical procedure for implanting biomaterials triggers the release of local mediators that bind to molecular pattern recognition receptors in the cells of the innate immune system [32]. This interaction activates the inflammasome, which leads to the expression of pro-inflammatory cytokines. [32]. This phase is associated with an acute inflammatory response, that plays an important role in the early adaptive immune response [32]. On the other hand, the biomaterials’ degradation products, and the surface modifications after implantation, activate interactions with the host immune system and the surrounding microenvironment [32]. The study of the interaction between innate and adaptive immune responses allows better understanding of the immune response to biomaterials, to obtain a balance for better results [32]. This interaction relies on the microenvironment surrounding the implant, which orchestrates tissue-specific innate immune responses followed by the activation of adaptive immune system responses. [39]. In the present study, the surgical method used to hold the PLGA dressings on the ulcerations did not provoke acute inflammatory responses or fibrosis.

Consistent with an earlier report by Chor et al. [12], other emerging therapies for oral mucositis using natural products, pharmaceuticals, growth factors, cells and biomaterials, demonstrated promising results in experimental models. In the present groundbreaking research work, our team explored the promising potential of autologous mesenchymal cells onto PLGA membranes to enhance re-epithelialization in experimental oral mucositis model. For example, Yonezawa [40] demonstrated that films of polyglycolic acid (PGA) adhered to the wounds of rabbits’ tongues using fibrin glue, which resulted in re-epithelialization. In addition, other studies that used PLGA also showed promising results [12]. Lee [41] demonstrated the effectiveness of electrospun PLGA membranes, whether infused with metformin (an antidiabetic) or not, as innovative dressings that significantly promoted the healing process of skin wounds in diabetic rats; Xu, [42] innovatively applied PLGA with mesenchymal cells to enhance bladder regeneration in experimental rabbit model.; Yoshimoto [43] successfully utilized PLGA for advancing periodontal tissue regeneration, demonstrating its effectiveness. And Hong [44], utilized PLGA for corneal regeneration, exhibiting its versatility in medical applications.

In recent clinical studies, Badr [13] utilized natural products such as honey and olive oil to treat chemotherapy-induced oral mucositis (OM) in children with leukemia. Serageldin [14] applied Metformin to address OM in non-diabetic breast cancer patients. Shah [15] explored the use of zinc for preventing OM in children with cancer who were undergoing intensified chemotherapy. Morsy [17] employed topical Omega-3 nanoemulgel for the treatment of radiotherapy-induced OM in patients with head and neck cancer. Additionally, Colella [5] recently reported a range of therapies, including medications, which demonstrated significant reductions in both the severity and incidence of OM. Those emerging therapies, and others that will shortly come, will help cancer patients to overcome the adverse symptoms of treatment, such as, leukopenia, weight loss and oral mucositis lesions.

Herein, our group examined the effects of 5-FU-induced immunosuppression and the initial regeneration process of oral mucositis lesions using hamsters models. Our objective was to explore bioengineering principles to develop a novel device that incorporates autologous cells. This innovative approach holds significant therapeutic potential, as it utilizes the host’s mesenchymal cells and nanotechnology tools to design a scaffold that mimics the organism’s microenvironment. [28]. Due to the complexity of the procedures that involved the animals, a restricted number of animals were chosen for this innovative preliminary approach. The procedures include: the isolation of autologous cells from the hamster cheek pouch to expand and seed in the PLGA membrane to proliferate; the development of chemotherapy-induced oral mucositis ulcerative lesions, and the application of autologous cell-seeded membrane as a dressing onto the ulcerative lesions. To our knowledge, this is the first-in-animal preliminary study, which used a biocompatible electrospun membrane with the addition of autologous cells for ulcerative oral mucositis regeneration. Although, the results are promising and have great potential for future applications, the low number of animals used may hinder the generalizability of the results. In view of the good results, we are encouraged to keep working with pre-clinical studies to demonstrate long-term outcome data. Our team anticipated promising results, as shown here, with the potential to be translated from the bench to the bedside in future clinical studies.

## MATERIALS AND METHODS

### *In vivo* experiment

Golden Syrian hamsters were purchased from Oswaldo Cruz Foundation-Rio de Janeiro, Brazil. Animals at six weeks of age were maintained at the Institute of Biological Science’s biotherium of the Federal University of Rio de Janeiro, UFRJ-Brazil, in environmental, nutritional, and health-controlled conditions, according to “The Guide for Care and Use of Laboratory Animals” (DHHS Publication No (NIH) 85-23, Office of Science and Health Reports, Bethesda, MD 20892-available at: http://www.nih.gov). The Ethics Committee on the Use of Animals in Scientific Experimentation at the Institute of Biological Science at Federal University of Rio de Janeiro, registered the study in the National Council for Animal Experimentation Control (CONCEA-Brazil). Therefore, they certified the use of hamsters in the present study (Protocol No. 003/15, approved on 15 April 2015). Animals between 120 and 205g (*n* = 10) were included in the present study. The animals were kept in appropriate cages containing three animals/cage, lined with sterile shavings, under constant temperature (23 ± 2°C), in the standard light/dark cycle (12/12 h), with unrestricted access to water and feed. After the experiments animals were euthanized according to the “American Veterinary Medical Association Guidelines on Euthanasia”, 2007 (available at http://www.nih.gov).

#### Leukogram and weight

The 5-FU-induced immunosuppression was monitored through peripheral blood leukogram and weight of 10 animals, performed before 5-FU administration (*n* = 5/group) or saline solution (SS) (day 1, Figure 6 - *n* = 5/group) and also on the fourth day after 5-FU or SS administrations (day 4 - Figure 6). To perform the leukogram, 50μL of peripheral blood was collected from the paw vein, through puncture with a needle (13 × 0.45 mm) after syringe treatment with sodium heparin by rinsing (Hepamax-S - sodium heparin 5000 IU/mL, Blau Farmacêutica S.A.). The global white blood cell count was determined by optical microscopy and counting in a Neubauer chamber. The leukogram was obtained by averaging two counts in different samples of suspensions of 5 μL of blood diluted in 95 μL of Türk’s hemolytic solution (dye for leukocytes).

Another clinical parameter considered along with immunosuppression was the animals’ weight before and after 5-FU or SS infusions, which was recorded using an electronic LCD digital weight (Figure 6) before any procedure that could generate stress in the animals, such as the scratch on the jugal mucosa of the animals for oral mucositis lesions development. The weights of the two populations of animals were recorded before 5-FU or SS administrations. These values were paired with the values of the weight obtained 4 days after 5-FU or SS infusions, and compared before the scratch procedure for OM development (Figure 6).

#### 5-Fluorouracil-induced OM ulcerations

To produce OM lesions, the animals were placed in a small containment (EB 285G Containment for rats – Insight equipment, São Paulo - Brazil) with openings on the lateral sides and bottom (Supplementary Figure 1) for breathing. One of the openings at the bottom was used to apply the first infusion of 5-Fluorouracil (5-FU/ Eurofarma) (80 mg/Kg - Figure 6), in the intraperitoneal region. On the second day of the protocol (Figure 6), the animals received the second infusion of 5-FU (60 mg/Kg) inside the small containment. On the third day of the protocol, animals did not receive 5-FU infusion. On the fourth day of the protocol, animals were anesthetized with ketamine (80 mg/kg) and xylazine (60 mg/kg) to receive a scratch on the right side of their cheek pouch, which was made using the tip of a needle of a 1mL syringe (Figure 6). The scratches were made horizontally and vertically in the same place of the jugal mucosa. On the same day of scratch, animals received the third 5-FU infusion (60 mg/kg). On days 5th and 6th of the protocol (Figure 6), animals were under observation and resting for ulcer formation. On the 7th day of the protocol, OM grade 4 (ulcerative lesions) were established (Figure 6).

### *In vitro* experiments

#### Primary fibroblasts proliferation

**Figure 7:**
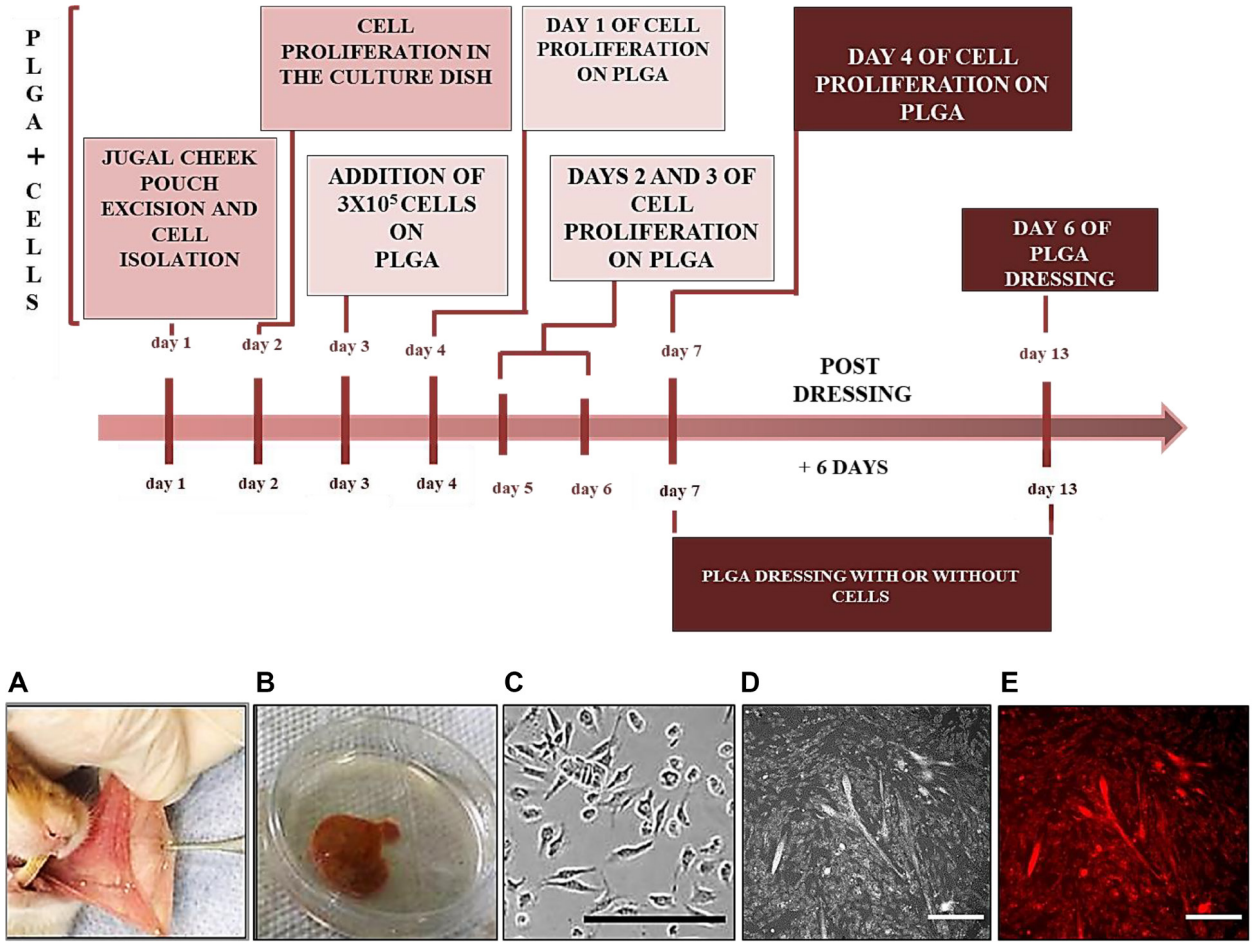
Timeline diagram showing cell isolation and proliferation on PLGA. Day 1, excision of the jugal cheek pouch and cell isolation (**A**, **B**); day 2, cell proliferation in the culture dish, such as represented by the cells in an amplified image in (**C**) (Nikon/ ECLIPSE Ti-S/objective 20X); day 3, addition of 3 × 10^5^ cells on the PLGA membrane; days 4, 5, 6, 7, the cells were proliferating on the PLGA membrane (**D**, **E** - Cell Tracker-red/Molecular Probes Life Technologies/lot 1756851/Nikon/ECLIPSE Ti-S/objective 20X); day 7, the PLGA dressing with or without cells were applied to the ulcerative lesions as dressings for 6 days; day 13, the dressing was removed and the ulceration region was excised for optical microscopy analysis. Scale bars: 100 µm.

Before the first 5-FU administration, the left side of hamsters’ cheek-pouch was excised after intraperitoneal injection of ketamine (80 mg/kg) and xylazine (60 mg/ kg) for primary fibroblasts isolation. Day 1 in the timeline diagram is represented by the macroscopic views of the cheek pouch (Figure 7A), and the excised cheek pouch (Figure 7B) of the animal. The excised cheek pouch was washed with povidone-iodine for 5 min. Next, rinsed with a saline solution 1 min, and after with nystatin (100.000 UI/Laboratorio Teuto S/A-Brasil) for 5 min. Finally, it was immersed in sterile saline solution for 5 min. Tissues were sectioned into small pieces under a laminar flow hood (Vertical laminar flow-Pachane LTDA, model PA 320, No. 03201. Pirasicaba, Brasil); immersed in collagenase type 1 solution (1 mg/mL-Gibco/Life Technologies) in culture medium (100 mL) inside an incubator at 37°C under agitation for 3 h and inactivated using fetal bovine serum (10%-Vitrocell/lot 014/18). Finally, the cells were isolated by filtration (cell strainer 100 μm/Sigma-Aldrich) and centrifugation (700 g) of the supernatant. The pellet was suspended in 1 mL low glucose DMEM culture medium, supplemented with 10% fetal bovine serum, 100 IU/mL penicillin, 100 mg/mL streptomycin and 0.01 mg/mL amphotericin (Sigma Aldrich, St. Louis, MO, USA). Next, cells were plated in a 25 cm^2^ culture bottle containing 4 mL culture medium, and kept in an incubator at 37°C in a 5% CO_2_ atmosphere. Culture medium was changed every 48 h after three washes with PBS, and stored at 37°C in a 5% CO_2_ atmosphere for 4 days (Figure 7C depicts cells in the culture dish).

#### PLGA membranes with cells

The membranes were covered with 100 µL of mouse collagen solution (Sigma-Aldrich) 10% (*v*/*v*) in serum-free DMEM. Next, the membranes were incubated in the laminar flow for 1 h, washed with PBS and dried in the laminar flow for another 1 hour. Next, the PLGA membranes were seeded with 3 × 10^5^ cells in 100 µL of complete DMEM and incubated over 3 days for cell adhesion and proliferation (Figure 7). Four days after cell proliferation (day 7 in the timeline diagram - Figure 7) the membranes (Figure 7D, 7E) were prepared for optical microscopy analyses (Nikon/ECLIPSE Ti-S/objetiva 20X) to confirm cell proliferations on PLGA. Figure 7D depicts PLGA membrane with cells and figure E is the same image of D, which was colored with a fluorescent CellTracker red to monitor the location of the live cells on to the membrane (*Cell Tracker-red/Molecular Probes Life Technologies*/lote 1756851).

#### PLGA membranes onto the OM lesions

On the 7th day of the protocol (Figure 6) the PLGA membranes with the addition of cells were ready to be applied on to the ulceration (first day of treatment - day 0 - Figure 6) and remained for 6 days. At the same time, the membrane without cells was applied onto the ulceration of another animal. The membranes were fixed after animals’ anesthesia, using a sterilized shirt button over the membrane (Supplementary Figure 2) [45]. The cellularized part of the membrane was applied on the ulceration, and the button was fixed on the other side of the membrane. The button was sutured using nylon (Nylon 5-0 c/24 1,5 Cm-*Shalon*) (Supplementary Figure 2). The suture knot was fixed on the animal’s face (Supplementary Figure 3). After the surgical procedure, the animals were kept in the Laboratory until they started to move. After that, the animals were transferred to their cages, containing only one animal in each cage. After six days of PLGA dressing fixation, (the 13th day of the protocol in the timeline diagram-Figure 7), the animals were anesthetized for surgical excision of the ulceration area for histomorphological analysis. Next, the animals were euthanized, by inhaling CO_2_ followed by decapitation using a guillotine. This is a first-in-animal model protocol for OM regeneration, and it was registered for patent request at the Industrial Property of National Institute, Brazil (Process number: BR 10 2023 027509 5), by *Núcleo de Inovação Tecnológica das Unidades de Pesquisa do MCTI/RJ-NITRIO* at the Brazilian Center for Research in Physics*,* Brazil*.*

### Sample preparation for morphological analyses

#### Light microscopy

The ulcer samples excised after material implantation were fixed in 4% paraformaldehyde in PBS, pH 7.4 at 4°C for 2 h. Next, washed in water, dehydrated in serial solutions of ethanol (from 30% to 100% twice for 20 min), clarified in xylene (2 baths of 30 min), and embedded in paraffin. Sections of 5 μm-thick were performed on a rotary microtome (Leica Microsystem RM 2125^®^, Wetzlar, Germany) and collected on glass slides. Dried histological sections were stained with hematoxylin-eosin (HE). The samples were analysed by light microscopy (Eclipse E800 light microscope, NIKON, Japan).

#### Immunohistochemistry

Paraffin sections were collected on silanized histological slides (Sakura Finetek, Staufen, Germany) for immunohistochemistry. After adhesion, histological sections were dewaxed in xylene and hydrated. The samples were washed with 50 mM ammonium chloride solution, in phosphate buffered saline (PBS) pH 8.0, for 15 min, to block free aldehyde residues and washed in PBS; permeabilized with Triton X100 (0.5%) in PBS for 15 min, followed by a bath containing 0.3% hydrogen-peroxide in methanol to inhibit endogenous peroxidase for 15 min, in the dark. After washing with PBS, pH 7.4, sections were submitted to heat-mediated antigen retrieval in either the microwave (potency 800 W) or steamer, according to the antibody used (Table 3). After cooling, the histological sections were incubated with PBS containing 5% bovine serum albumin (BSA), normal 5% goat serum (1 h), and primary antibodies (Table 3), in a humid environment, at room temperature. Next, the sections were kept in a humid environment for 20 hours in the refrigerator. Afterwards, the sections were washed in a PBS solution containing 0.25% Tween-20 (PBS-Tween), followed by incubation with the secondary antibody conjugated to peroxidase (Envision TM Dual link systemHRP-cat. No. K4601- Dako, CA, USA) for 1 h, followed by washes in PBS-Tween. Peroxidase was developed with the chromogenic substrate diaminobenzidine (Liquid DAB, Dako, cat. No. K3468), followed by washes in PBSTween and distilled water and sections were dehydrated in ethanol and clarified in xylene. Then sections were mounted with Entellan^®^. Negative controls were performed by incubating the histological sections with non-immune rabbit or mouse serum or with the antibody diluent in place of the primary antibody. The samples were analysed by light microscopy (Eclipse E800 light microscope, NIKON, Japan).

**Table 3:** Characteristics of antibodies used and antigenic recovery in the immunohistochemistry assays

Antibody	Manufacturer	Antigenic recovery	Dilution
α-SMA	Dako, polyclonal rat, cat.# GKX-5010	Steamer 20 min, Citrate Buffer 0.01 M pH 6.0	1: 100
F4/80	AbCam, CA, USA, polyclonal rabbit, cat.# ab9535	Microwave—3 min 3 x, Citrate Buffer 0.01 M pH 6.0	1:100

### Statistical analysis

Two leucograms were performed for each animal, and the average of the readings was considered the final result before and after 5-FU infusion or saline solution, for statistical analysis. Wilcoxon test was used for comparisons between independent leukograms of the same population, paired to be analysed in different times. Mann Whitney, was used for comparisons between independent leukograms from independent treatments, in different populations of animals at the same time.

The weights of the two independent populations of animals were recorded before and after 5-FU or SS administrations for statistical analysis. Paired *t*-test was used for comparisons between independent weights in the same population of animals, pared to be analysed in different times. Graph pad Prisma version 5.0 was used for statistical analysis, and the *p* < 0,05 value was considered the reference of the statistical hypothesis for leucogram and weight.

## CONCLUSIONS

In the present study, leukopenia and weight loss were induced by 5-Fluorouracil infusions confirming the immunosuppression in hamster’s model. In virtue of these parameters, OM ulcerations were established in the jugal mucosa of the animals. The combined tools of nanotechnology and biology allowed the design of the present innovative approach in the bioengineering area. The application of electrospun PLGA membrane with the addition of autologous cells for OM ulceration, revealed a promising regenerative result. This potential result will boost other studies in the tissue engineering of the buccal mucosa in a near future.

## SUPPLEMENTARY MATERIALS


