# Supplementary material for: Leukopenia, weight loss and oral mucositis induced by 5-Fluorouracil in hamsters’ model: A regenerative approach using electrospun poly(Lactic-co-Glycolic Acid) membrane
Source: Oncotarget. 2025 Feb 18;16:103–17. doi: 10.18632/oncotarget.28685 (PMC11837862; doi:10.18632/oncotarget.28685)
Supplement: Supplementary file 1 [file oncotarget-16-28685-s001.pdf]

Leukopenia, weight loss and oral mucositis induced by 5-Fluorouracil in hamsters' model: A regenerative approach using electrospun poly(Lactic-co-Glycolic Acid) membrane

SUPPLEMENTARY MATERIALS

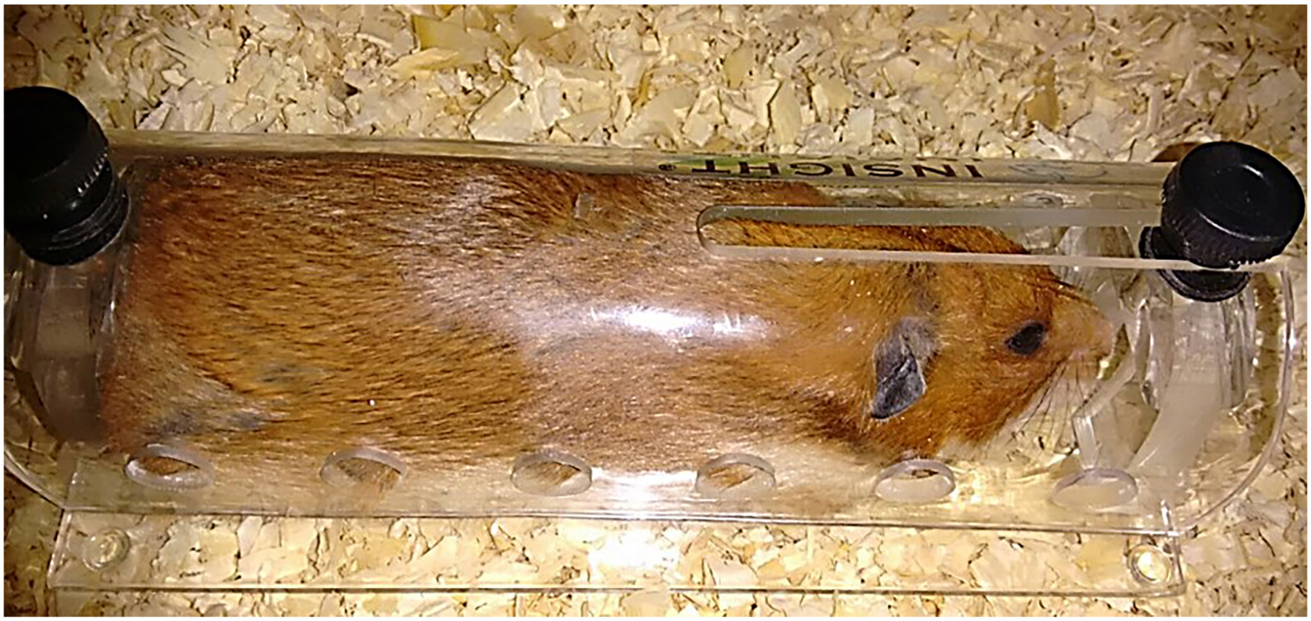

Supplementary Figure 1: Small containment (EB 285G Containment for rats – Insight equipment, São Paulo - Brazil) with openings on the lateral sides and bottom for breathing and to contain the animal for chemotherapy infusions.

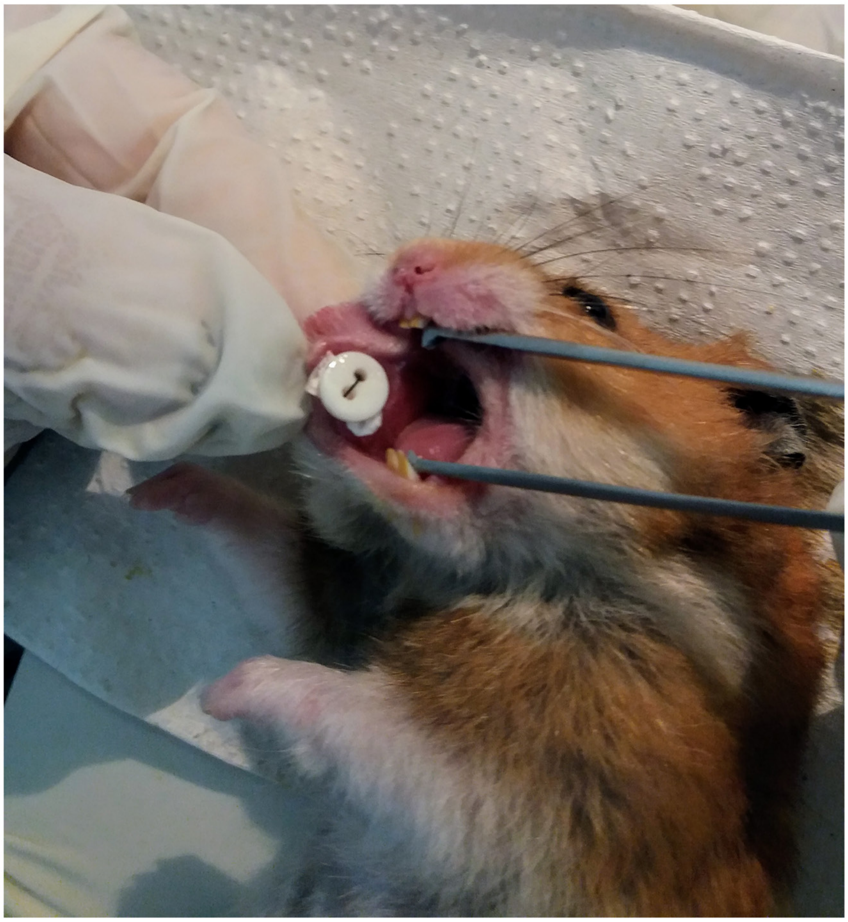

Supplementary Figure 2: Photo showing the ulceration area covered by the biomaterial (PLGA), which was fixed by a button with suture.

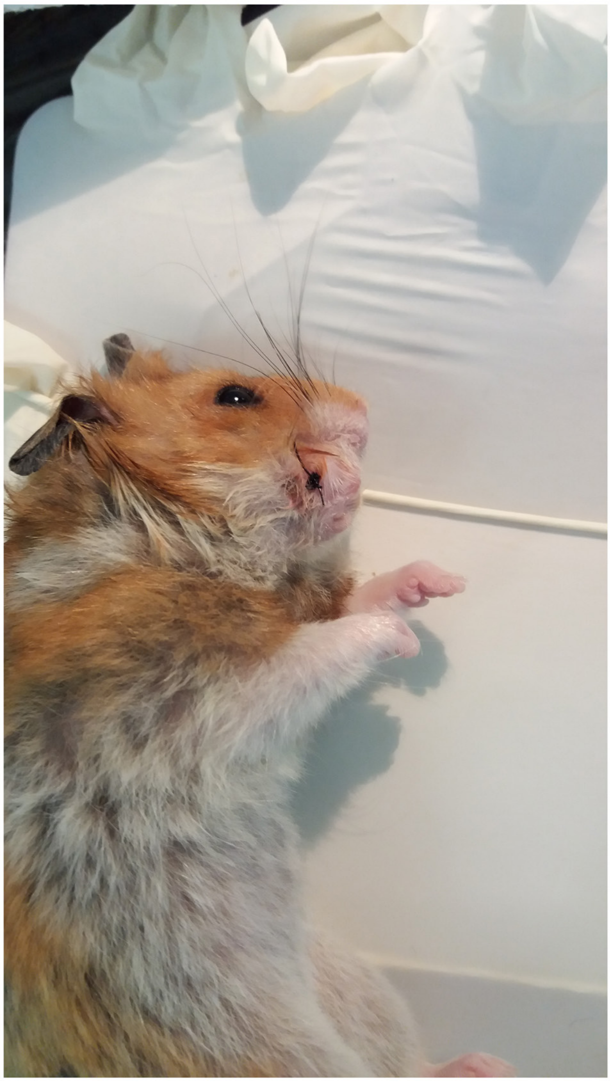

Supplementary Figure 3: Photo showing the suture knot on the face of the animal, which fix the button through the PLGA membrane and the jugal mucosa of the animal.
